# Полногеномные ассоциативные исследования распространения пороков развития и других селекционно значимых качественных признаков у потомства хряков крупной белой породы российской селекции

**DOI:** 10.18699/VJ20.612

**Published:** 2020-03

**Authors:** А.А. Траспов, О.В. Костюнина, А.А. Белоус, Т.В. Карпушкина, Н.А. Свеженцева, Н.А. Зиновьева

**Affiliations:** Федеральный научный центр животноводства – ВИЖ им. академика Л.К. Эрнста, пос. Дубровицы, Подольский городской округ, Московская область, Россия; Федеральный научный центр животноводства – ВИЖ им. академика Л.К. Эрнста, пос. Дубровицы, Подольский городской округ, Московская область, Россия; Федеральный научный центр животноводства – ВИЖ им. академика Л.К. Эрнста, пос. Дубровицы, Подольский городской округ, Московская область, Россия; Федеральный научный центр животноводства – ВИЖ им. академика Л.К. Эрнста, пос. Дубровицы, Подольский городской округ, Московская область, Россия; Федеральный научный центр животноводства – ВИЖ им. академика Л.К. Эрнста, пос. Дубровицы, Подольский городской округ, Московская область, Россия; Федеральный научный центр животноводства – ВИЖ им. академика Л.К. Эрнста, пос. Дубровицы, Подольский городской округ, Московская область, Россия

**Keywords:** marker assign selection, quantitative trait loci, SNP-chips, genome-wide association studies, malformations, маркер-зависимая селекция, количественные признаки, SNP-чипы, полногеномные ассоциативные исследования, пороки развития свиней

## Abstract

Выявление областей генома, прямо или опосредованно связанных с признаками пороков развития у домашних свиней, может способствовать идентификации генетических мишеней, используемых в качестве биомаркеров индивидуальных особенностей формирования экстерьера, их метаболического статуса, а также подверженности генетическим заболеваниям. Такие исследования напрямую связаны с повышением экономической эффективности, поскольку позволяют выявлять и исключать из селекционного процесса животных-носителей нежелательных генов, фенотип которых может не проявляться. В данной работе проведен поиск подобных целевых генов и геномных регионов с помощью полногеномных ассоциативных исследований (GWAS) с использованием ДНК-чипов PorcineSNP60K BeadChips (Illumina, San Diego, USA). Проанализировано 48 хряков свиней крупной белой породы селекционно-гибридного центра «Знаменский» Орловской области по 21 недостатку экстерьера и дефектам развития у 39 153 их потомков. Расчеты производили по линейной модели смешанного типа в программном пакете GEMMA. Из изначального сета в 61 000 SNP были отобраны 36 704 полиморфных SNP, в которых найдены 24 полиморфизма, входящих в 11 генов (P < 0.1), статистически значимо коррелирующих с признаками аномалий развития в геноме свиней, такими как атрезия анального отверстия (ARMC7, FANCC, RND3, ENSSSCG00000017216), проблемы с конечностями (PAWR, NTM, OPCML, ENSSSCG00000040250, ENSSSCG00000017018) и тремор поросят (RIC3, ENSSSCG00000032665). Также была выявлена коэкспрессия генов NTM, OPCML и RND3, участвующих в регуляции клеточной адгезии. Проведенная работа подтвердила актуальность применения подобного подхода в полногеномно-ассоциативных исследованиях для детектирования единичных SNP, связанных с отдельными признаками, даже для небольших выборок.

## Abstract (en)

Identifying genome regions that are directly or indirectly associated with developmental defects and malformations in domesticated pigs can help identify genomic traits used as biomarkers of the structural and functional composition of the body, their metabolic status and genetic diseases as well. Such studies are directly related to the improvement of the economic efficiency, as they allow identification and exclusion of defect animals, who may carry target genes not appearing phenotypically, from the breeding process. In the current work, we have searched for these kind of target genes and genome regions with conducting the genome-wide association studies using PorcineSNP60K BeadChips (Illumina, San Diego, USA). A total of 48 boars of a large white breed of the nucleus farm “Znamenskoe” were analyzed for 21 traits of indicated shortcomings of the exterior and defects of development in 39,153 their offspring. Calculations were made using a mixed type linear model in package GEMMA. In this study, we selected only 36,704 polymorphic SNPs from an initial 61,000-strong SNP set. After GWAS, we obtained 24 alleles in 11 corresponding genes (P < 0.1) in the genome of pigs, which are significantly correlated with traits of developmental abnormalities such as anal atresia (ARMC7, FANCC, RND3, ENSSSCG00000017216), limb problems (PAWR, NTM, OPCML, ENSSSCG00000040250, ENSSSCG00000017018) and tremor of piglets (RIC3, ENSSSCG00000032665). Also, co-expression of the NTM, OPCML and RND3 genes was revealed. This study confirms the relevance of using the single SNP detection according to the single trait approach in associative studies, even for small sample numbers.

## Введение

В свиноводстве болезни приводят к большим экономи- ческим потерям не только из-за затрат на медикаментоз- ное лечение, но и вследствие снижения продуктивных показателей больных животных. На данный момент у домашних свиней насчитывается более 130 болезней как наследственной, так и инфекционной этимологии (https:// thepigsite.com/disease-guide). Расширение знаний о причинах болезней позволит нивелировать их влияние на организм благодаря более совершенным программам разведения (Boddicker et al., 2012). Отклонения от нор- мального развития могут затрагивать различные органы и системы, ухудшая физическое состояние животного или даже приводя к смерти. Анатомические аномалии или дефекты, вызванные генетическими или экологическими факторами, встречаются по крайней мере у 1 % новорож- денных поросят. В отдельных стадах такие аномалии мо- гут встречаться с достаточно высокой частотой, приводя к значительным экономическим потерям (See et al., 2006).

Одна из стратегий снижения экономических потерь, обусловленных наследственными болезнями, – это выяв- ление и исключение из разведения животных, генетически чувствительных к таким заболеваниям. Например, уже с начала 1990-х гг. селекционеры используют технологию маркерной селекции для выявления нежелательных ал- лелей гена HAL, вызывающего синдром стресса свиней, и гена RN, обусловливающего дефект «кислого мяса» (Salas, Mingala, 2017). Дополнение индексов племенной ценности (EBV) информацией, полученной на основании анализа непосредственно генотипа животного, делает возможным создание нового типа индекса – GEBV (genetic evaluation breeding value), характеризующегося более высокой точностью. Таким образом, дополнение традиционных методов оценки молекулярно-генетическими данными стало шагом вперед в направлении повышения интенсивности искусственного отбора (Племяшов, 2014). Выявление молекулярных маркеров, ответственных за желательные фенотипические эффекты, облегчает селекционный процесс и ускоряет получение прибыли в производстве (Ernst, Steibel, 2013). Исследования ассоциаций ДНК-маркеров в свиноводстве привлекают внимание ученых как в нашей стране (Долматова, Сковородин, 2010), так и за рубежом (Bruun et al., 2006; Ciobanu et al., 2011). Понимание генетических механизмов, ответственных за конкретные генетические аномалии, поможет производителям племенной продукции в разработке методов отбора, поскольку разные типы маркеров в геноме отвечают за разные фенотипические признаки. Так, с помощью MAS (маркер-зависимая селекция) можно проводить не только выбраковку, но и целевой отбор животных, устойчивых к заболеваниям. К примеру, отбор индивидуумов с отсутствием рецепторов адгезии E. coli на поверхности кишечника (K88) позволяет получить от них потомство, устойчивое к колибактериозу (Nyachoti et al., 2012). Рас- крытие механизмов появления генетических аномалий поможет производителям в разработке методик отбора животных с «желательными» генотипами.

Современные методы полногеномных исследований (детекция SNP, полногеномное секвенирование) находят применение в выявлении генетических факторов и, как следствие, в понимании биологических процессов, лежащих в основе развития экстерьера у свиней. С учетом возможного сцепленного наследования и коэкспрессии соседних генов, детекции отдельных SNP может быть недостаточно для детального изучения комплексных признаков заболеваний или резистентности к ним. Включение в селекционные программы ДНК-маркеров QTL в качестве дополнительного критерия позволяет повысить точность оценки племенной ценности животных в аспекте их продуктивности, с учетом потенциального носительства генетических дефектов или наличия резистентности к ряду заболеваний (Sermyagin et al., 2016, 2018).

## Материалы и методы

Исследования проводили на хряках крупной белой по- роды и их потомках, разводимых в ООО «Знаменский селекционно-гибридный центр» Орловской области. Было произведено полногеномное генотипирование хряков (n = 48) с использованием ДНК-чипа средней плотности Porcine SNP60BeadChip (Illumina Inc., США). Контроль качества геномных данных выполняли в программном пакете Plink 1.9. Использовались параметры качества генотипирования 90 % для одного SNP (geno 0.1), для одного образца (mind 0.1), а также для частот минорных аллелей не более 0.5 % (maf 0.05) (Purcell et al., 2007). Всего филь- трацию прошли 36 704 полиморфных SNP.

База фенотипов потомков хряков была получена из ООО «Знаменский селекционно-гибридный центр». База данных содержала 31 нежелательный показатель для каж- дого животного. У потомков хряков (n = 39 153) рассчиты- вали частоту встречаемости изучаемых признаков путем деления числа носителей фенотипического показателя на общее число потомков. Для проверки гипотезы о нормальном распределении использовали критерий согласия Пирсона (χ^2^ для уровня значимости 0.05) с последующей нормализацией данных в пакете bestNormalize для языка R

(Peterson, 2017). В итоге мы получили 21 нормализированный показатель, характеризующий пороки экстерьера и другие нежелательные качественные показатели: крипторхизм (CR), недоношенность (AF), атрезия ануса (AA), черные и серые пятна на шкуре (BD, GD), недовес при рождении (LW), несоответствие породе (WB), общий вес при рождении (CW), проблемы с пищеварением (DP), гермафродитизм (HM), наличие пупочной и паховой грыж (CH, UH), низкий материнский индекс (LSI), пониженное либидо у хряков (LL), несовершенный эпителиогенез (SL), низкая интенсивность роста (SG), некачественное семя у хряков-производителей (LSQ), синдром спастического тремора поросят (TP), уродства (UP), искривление конечностей (CL) и аномалии копыт (HA).

Полногеномный анализ ассоциаций (GWAS) выполняли в программном пакете GEMMA, используя линейную модель смешанного типа для частот встречаемости:

**Formula:**
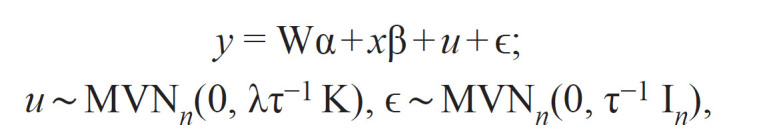
1.

где y – ковариантный признак (наличие/отсутствие забо- левания или другого изучаемого качественного признака в виде бинарных значений 0 или 1); W = (w_1_, … , w_c_ ) – матрица коварианс (фиксированные эффекты), α – пере- хватывающий коэффициент, x – маркерные генотипы; β – эффект маркера, u – случайные эффекты; ϵ – ошибки; τ^−1^ – дисперсия остаточных ошибок; λ – отношение между двумя компонентами дисперсии; K – матрица родства, соотнесенная с идентификационной матрицей I_n_; MVN_n_ – многомерное нормальное распределение.

Матрица родства рассчитывалась по формуле (в данном случае X – матрица n × P генотипов)

**Formula:**
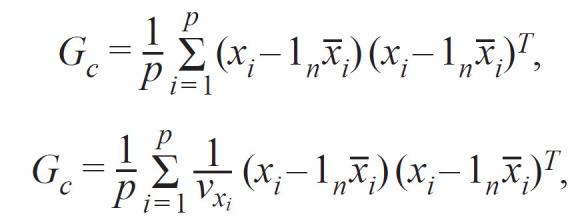
2.

где x_i_ – каждая i-я колонка с генотипами каждого i-го SNP; x_i_ – среднее для образца; v_x_i__ – варианса для каждого образца i-го SNP; 1n – вектор для каждого (n × 1) первого образца.

В частности, SNP с меньшей малой частотой аллелей имеют тенденцию оказывать больший эффект (который обратно пропорционален его вариации в генотипе), и в подобном случае выбирается первая модель матрицы (Zhou, Stephens, 2012). Проверка альтернативной гипотезы H_1_: β ≠ 0 и H0: β = 0 для каждого SNP, в свою очередь, проводилась по трем наиболее распространенным статистическим тестам (Wald, likelihood ratio test или score). В данной работе пакетом GEMMA была автоматически получена оценка максимального правдоподобия параметров λ и β (Maximum Likelihood Estimate) для дальнейшего вычисления соответствующего значения P (Zhou, Stephens, 2012). Фильтрация дисперсионных компонент λ была проведена с пороговым значением P < 1e–10. Для подтверждения достоверного влияния SNP и определения значимых регионов в геноме животных были применены тесты Бонферрони (BFR) с пороговым значением P ≤ 0.1 (P < 2.86 × 10–6) и ожидаемой долей ложных отклонений B. Efron по количеству SNP отдельно для каждого признака (Benjamini, Hochberg, 1995). При вычислении скорректированных индексов Q использовался список P-значений, полученных в результате одновременного тестирования многих гипотез (Wald, likelihood ratio или Score) (Benjamini, Hochberg, 1995). Q-значения измеряли долей ложно-позитивных индексов P в случае прохождения порогового интервала (Storey et al., 2017). В данном исследовании основным критерием был установлен пороговый интервал P < 0.1.

Для поиска генов, ассоциированных с изучаемыми признаками, использовали данные VEP (variant effect predictor) (McLaren et al., 2016). Для визуализации значений P и геномного контроля λ были построены Manhatten и QQ графики в пакете qqman с помощью языка програм- мирования R (Storey et al., 2017; Turner, 2017). Идентифи- кацию генов и их функциональную аннотацию осущест- вляли по базе взаимосвязей STRING (https://string-db.org/cgi/input.pl). Матрицы гаплотипов были построены посредством программного пакета Haploview (Barrett et al., 2005).

## Результаты

По результатам исследования были установлены значимые (согласно критериям BFR, с пороговым значением P < 0.1) ассоциативные связи для трех из 21 проанализированного качественного показателя хряков-произ- водителей: атрезия ануса, AA; синдром спастического тремора поросят, TP; аномалии копыт, HA. Пять SNP с высокими значениями достоверности обнаружены для признака AA (P = 1.16e–06…3.68e–09), пять – для TP (P = 1.721e–06…1.24e–08) и четырнадцать – для HA (P = 1.766e–06…1.737e–09) (см. таблицу).

**Table 1.**
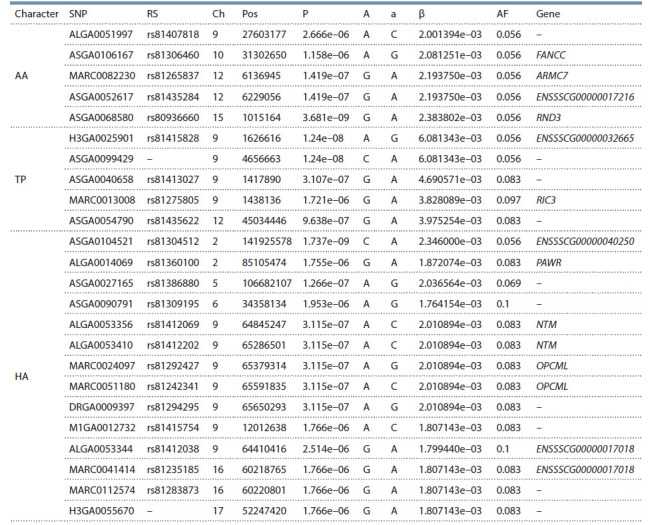
Significant associations of single nucleotide polymorphisms (SNP) associated with the assessed traits in piglets and their positions in the Sus scrofa genome Designations: SNP, single nucleotide polymorphism; RS, SNP identifier in the NIH dbSNP database; Ch, chromosome; Pos, position; P, validity; A, effector allele; a, reference allele; β, allele effect; AF, effector allele frequency; Gene, name of the gene housing the studied SNP.

Для AA и TP (λ ~ 1) уровень инфляции статистики был на номинальном уровне, а для всех признаков коэффициент геномного контроля был близок к единице, как показано на графиках квантиль–квантиль (QQ-график, рис. 1). Однако у HA выявлено наличие близкорасполoженных SNP, входящих в одинаковые группы генов со значительными превышениями P порога (ASGA0104521, P = 1.737e–09). Значимые нуклеотидные полиморфизмы были локализованы внутри отдельных генов. В ходе расчета LD между SNP с самыми высокими значениями P были отобраны полиморфизмы со значениями r^2^, наиболее близкими к 1 по отношению к ALGA0053356 (P = 3.115e–07, Pos 9:64845247). В результате в один блок с ним вошли ALGA0053410, MARC0024097, MARC0051180, DRGA0009397, что подтверждает ожидаемое функциональное родство отобранных SNP и генов, их включающих (рис. 2).

**Fig. 1.**
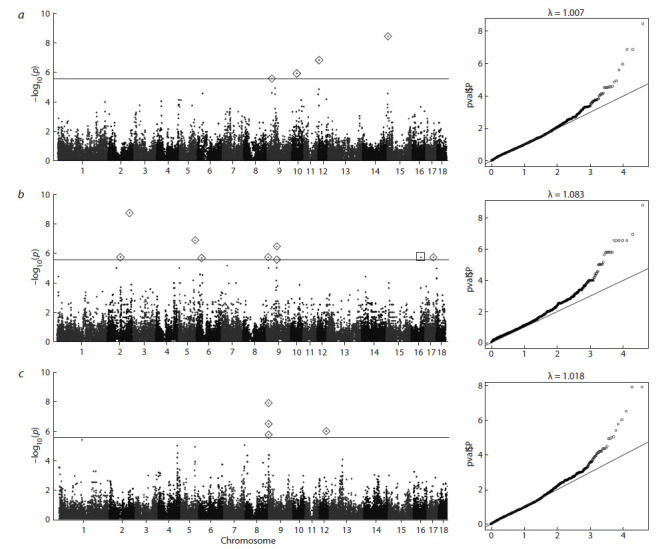
Manhattan plot illustrating GWAS (genome-wide association study, top-left) and corresponding probability distributions of confidence P (Q-Q graph) in piglets of the studied populations of large white pigs (Znamenskiy Breeding Center): a, anal atresia (AA); b, hoof problems (HA); and c, spastic tremor (TP). The solid line indicates the Bonferroni level (0.05). Reliable values satisfying the null Bonferroni hypothesis (BFR) are marked with diamonds.

**Fig. 2.**
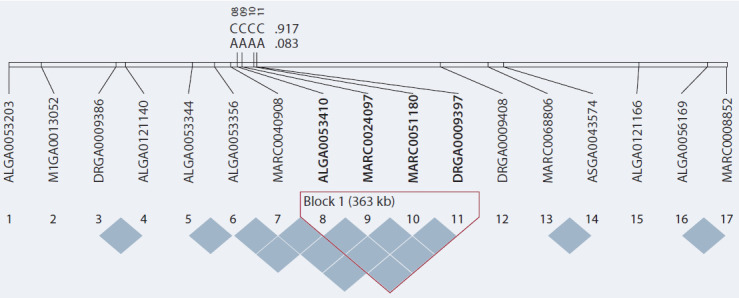
Haploview LD chart illustrating 17 SNPs with the highest r2 values of coupling between ALGA0053356 and four polymorphic variants – ALGA0053410, MARC0024097, MARC0051180, and DRGA0009397 to form a 363-kb haploblock in group HA.

Анализ SNP, статистически значимо связанных с пороками развития поросят крупной белой породы, выявил несколько генов, имеющих отношение к различным биологическим процессам. Так, гены ARMC7, FANCC участвуют в репарации ДНК и клеточном цикле. FANCC принимает участие в передаче анемии Фанкони, RND3 выступает как регулятор цитоскелетных структур клетки, препятствующих адгезии. UBAP2 функционирует в процессе убиквитинирования и может проявлять повышенную экспрессию в надпочечниках и лимфатических узлах. Ген PAWR является опухолевым супрессором, который селективно индуцирует апоптоз в раковых клетках через внутриклеточные и внеклеточные механизмы. RIC3 влияет на свертывание и сборку рецепторных субъединиц в эндоплазматическом ретикулуме и адгезию на поверхности клетки.

Гены NTM и OPCML экспрессируются совместно и находятся на соседних участках 9-й хромосомы: 58700168–58967505 и 59037716–59271936 п. н. соответ- ственно (www.ncbi.nlm.nih.gov/gene/100519556, www.ncbi.nlm.nih.gov/gene/100738337) (рис. 3). NTM способствует росту и адгезии на поверхности нейронов и тесно связан с родственным членом семейства, опиоидным связывающим белком-активатором клеточной адгезии OPCML.

**Fig. 3.**
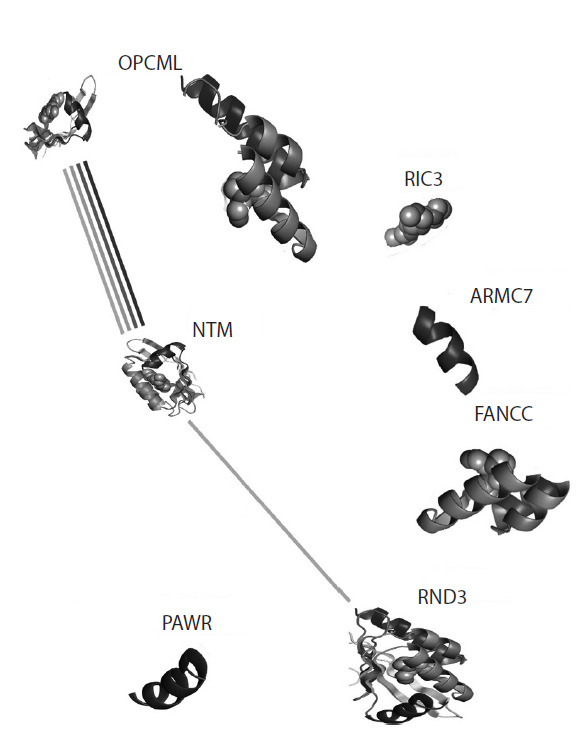
String diagram (https://string-db.org) illustrating the strong protein- mediated interaction of the coexpressed OPMCL and NTM genes, as well as their indirect effect on RND3.

## Заключение

Полученные нами данные могут быть использованы при разработке селекционных программ, направленных на элиминацию пороков развития и других нежелательных количественных и качественных признаков свиней, в том числе являющихся сложными признаками. Увеличение степени разрешения сканирования от 100 000 SNP и выше, а также увеличение размера выборки от нескольких со- тен животных и более сделает возможным выявление значительно большего количества SNP-кандидатов с высоким уровнем достоверности (P < 0.01), а также уменьшение «генетического шума» (false positive components). В итоге такой метод детекции позволит не только выявлять животных-носителей генов-кандидатов нежелательных признаков, но и создать дешевые тест-системы для их идентификации. Хряков-производителей, имеющих подобные генетические особенности, необходимо оценивать с помощью комплексных моделей расчета племенной ценности с учетом выявленных маркеров (GEBV) и выбраковывать в случае крайне низких показателей продуктивности, а их потомство исключать из воспроизводства.

Проделанная работа иллюстрирует необходимость про- ведения дополнительных исследований с использованием методов GWAS в аспекте характеристики популяций сельскохозяйственных животных по ДНК-маркерам и иденти- фикации комплексных генотипов, ассоциированных с се- лекционно значимыми признаками как пороков развития, так и продуктивных качеств. Данное направление крайне необходимо в современных условиях высокоэффектив- ного воспроизводства сельскохозяйственных животных.

## Conflict of interest

The authors declare no conflict of interest.
